# Impact of Psychiatric Comorbidity on Outcomes of Interventional Pain Procedures: A Retrospective Cohort Study

**DOI:** 10.7759/cureus.109090

**Published:** 2026-05-18

**Authors:** Paul Salib, Natasha Mehta, Joseph Mouhanna

**Affiliations:** 1 Pain Medicine, Larkin Community Hospital, South Miami, USA; 2 Pain Medicine, Larkin Community Hospital South, Hialeah, USA; 3 Pain Management, Larkin Community Hospital, South Miami, USA

**Keywords:** anxiety, chronic pain, depression, interventional pain procedures, psychiatric comorbidity

## Abstract

Background

Psychiatric comorbidities such as depression, anxiety, and post-traumatic stress disorder are common in patients with chronic pain and may adversely affect pain perception and treatment response. Their impact on outcomes following interventional pain procedures remains insufficiently characterized.

Objective

To assess the effect of baseline psychiatric comorbidities on clinical outcomes after interventional pain procedures.

Methods

This retrospective cohort study included 100 patients who underwent interventional pain procedures between 2023 and 2025 at a tertiary pain center. Patients were stratified by the presence or absence of documented psychiatric diagnoses. The primary outcome was the change in Numeric Rating Scale (NRS) pain score at three months. Secondary outcomes included functional improvement and changes in opioid consumption. Continuous variables were analyzed using Student’s t-test and categorical variables using chi-square testing, with significance defined as p < 0.05.

Results

Among 100 patients, 46 had psychiatric comorbidities, and 54 did not. Patients without psychiatric diagnoses demonstrated significantly greater reductions in pain scores compared to those with psychiatric conditions (mean reduction 3.4 vs. 2.1, t = 2.61, df = 98, p = 0.01). Functional improvement was significantly more common among patients without psychiatric comorbidities versus those with psychiatric conditions (72% vs. 48%, χ^2^ = 5.94, p = 0.01). Opioid reduction was also significantly more frequent in the non-psychiatric group (56% vs. 32%, χ^2^ = 5.76, p = 0.02).

Conclusions

Baseline psychiatric comorbidities are associated with diminished clinical improvement following interventional pain procedures. Integrating psychological assessment and multidisciplinary care may enhance treatment outcomes in this population.

## Introduction

Chronic pain affects approximately 20% of adults worldwide and remains one of the leading causes of long-term disability, healthcare utilization, and reduced quality of life [1,2]. Beyond its physical manifestations, chronic pain is increasingly recognized as a multidimensional condition influenced by biological, psychological, and social determinants. Psychiatric comorbidities, particularly depression, anxiety disorders, and post-traumatic stress disorder (PTSD), are highly prevalent among patients with chronic pain and contribute substantially to symptom severity, functional impairment, healthcare costs, and treatment resistance [3-5].

The relationship between chronic pain and psychiatric illness is bidirectional and complex. Depression and anxiety may amplify pain perception through alterations in descending inhibitory pain pathways, neuroinflammatory signaling, central sensitization, and maladaptive cognitive processing [6-8]. Conversely, persistent pain itself may worsen psychiatric symptoms by impairing sleep, mobility, social functioning, and overall quality of life [9]. Neuroimaging studies have demonstrated overlapping neural circuitry between affective disorders and pain processing, including abnormalities involving the anterior cingulate cortex, insula, amygdala, and prefrontal cortex [10].

Interventional pain management techniques, including epidural steroid injections, medial branch blocks, radiofrequency ablation, sacroiliac joint injections, vertebral augmentation procedures, and spinal cord stimulation, are commonly utilized for patients with refractory chronic pain conditions [11,12]. Although many patients experience meaningful pain reduction and functional improvement following these procedures, clinical outcomes remain heterogeneous and difficult to predict. Identifying patient-specific factors associated with treatment response has therefore become increasingly important in modern pain medicine [13].

Several prior investigations suggest that psychiatric comorbidities negatively influence outcomes after spine surgery, spinal cord stimulation, opioid therapy, and multidisciplinary rehabilitation programs [14-16]. Depression and anxiety have been associated with higher baseline pain intensity, greater opioid utilization, reduced procedural satisfaction, and poorer long-term functional recovery [17,18]. Pain catastrophizing and maladaptive coping strategies may additionally contribute to diminished treatment response through enhanced central pain amplification and behavioral avoidance patterns [19].

Despite growing recognition of the biopsychosocial model of pain, relatively limited literature has specifically evaluated the influence of psychiatric comorbidities on outcomes following common interventional pain procedures performed in routine clinical practice. Most available studies focus on isolated interventions or narrowly defined patient populations, limiting generalizability to broader pain management settings [20].

The present study aimed to evaluate the impact of baseline psychiatric comorbidities on clinical outcomes following interventional pain procedures in a real-world tertiary pain management population. We hypothesized that patients with documented psychiatric disorders would demonstrate less improvement in pain scores, functional outcomes, and opioid utilization compared with patients without psychiatric comorbidities.

## Materials and methods

Study design

This study was conducted as a retrospective cohort analysis of patients treated at a single tertiary pain management center. The research and data synthesis were performed at Larkin Community Hospital, South Miami and Hialeah, Florida, USA.

Study population

Medical records were reviewed for patients who underwent interventional pain procedures between January 2023 and January 2025.

Inclusion criteria

Patients were included if they met the following criteria: age ≥ 18 years, chronic pain duration greater than six months, underwent at least one interventional pain procedure, and had documented baseline and three-month follow-up pain scores.

Exclusion criteria

Patients were excluded if they had active cancer-related pain, acute traumatic pain, incomplete follow-up data, or less than three months of follow-up.

Psychiatric stratification

Patients were categorized based on documented psychiatric diagnoses in the electronic medical record. The psychiatric comorbidity group included patients with major depressive disorder, generalized anxiety disorder, PTSD, or bipolar disorder. The non-psychiatric group included patients without documented psychiatric diagnoses.

Interventional procedures

Procedures analyzed in this study included lumbar and cervical epidural steroid injections, medial branch blocks, radiofrequency ablation, sacroiliac joint injections, and spinal cord stimulator trials.

Outcome measures

The primary outcome was the change in Numeric Rating Scale (NRS) pain score from baseline to three months. Secondary outcomes included functional improvement and changes in opioid consumption, measured in morphine milligram equivalents.

Statistical analysis

Statistical analysis was performed using IBM SPSS Statistics for Windows, Version 27 (Released 2019; IBM Corp., Armonk, New York, United States). Continuous variables were summarized as means ± standard deviations and compared using an independent-samples Student’s t-test, with corresponding t-values and degrees of freedom reported where applicable. Categorical variables were expressed as frequencies and percentages and analyzed using the chi-square test. A two-tailed p-value < 0.05 was considered statistically significant.

Given the retrospective design, a formal sample size calculation was not performed. A convenience sampling approach was used, including all eligible patients during the study period. Multivariate regression analysis was not performed to avoid model instability and overfitting.

## Results

**Figure 1:**
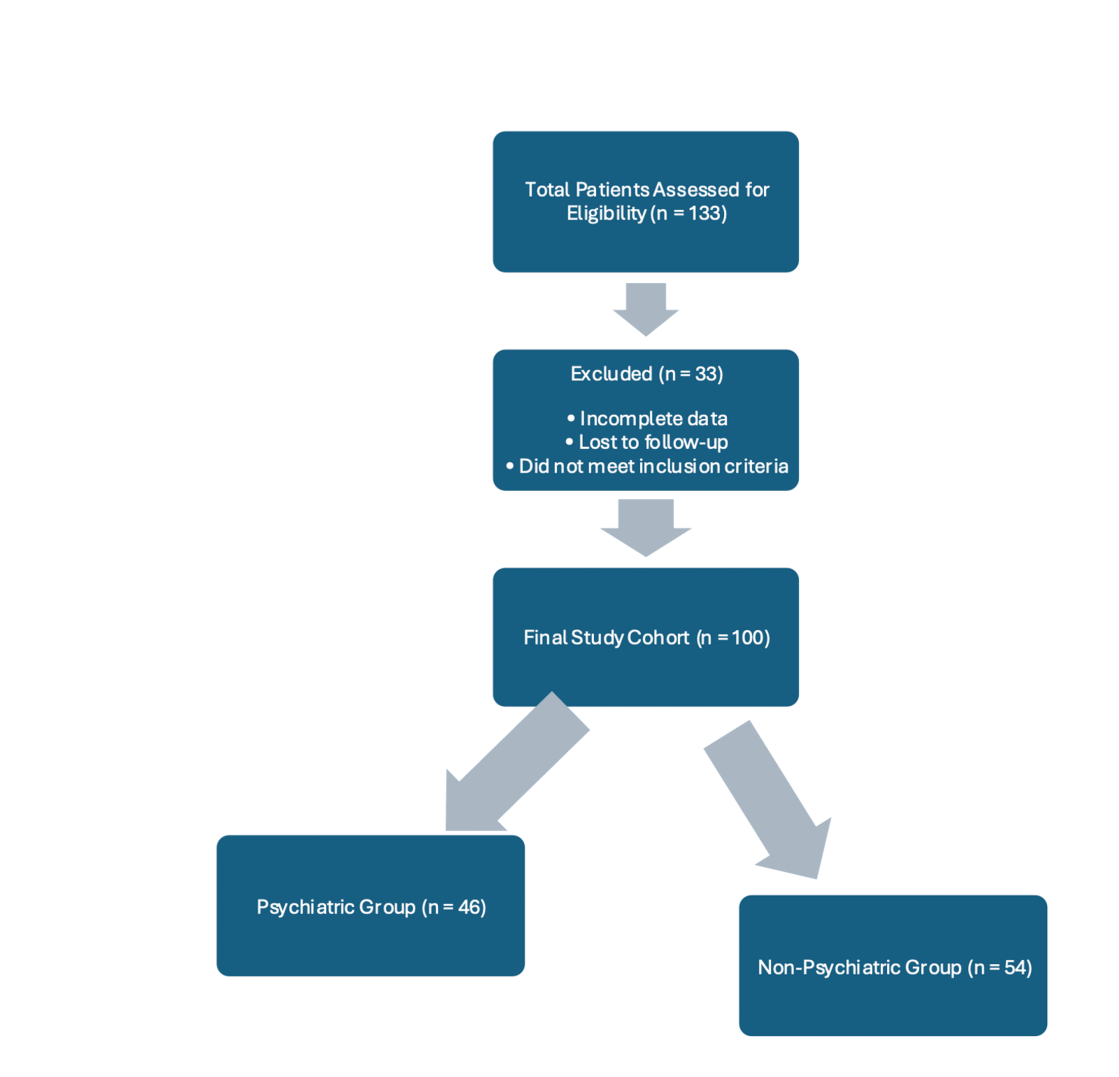
Study flow diagram illustrating patient selection and cohort allocation. A total of 133 patients were assessed for eligibility. Thirty-three patients were excluded because of incomplete data, loss to follow-up, or failure to meet the inclusion criteria. The final study cohort comprised 100 patients, of whom 46 had documented psychiatric comorbidities, and 54 had no psychiatric diagnoses.

A total of 133 patients were assessed for eligibility, of whom 33 were excluded due to incomplete data, loss to follow-up, or failure to meet inclusion criteria. The final study cohort consisted of 100 patients, including 46 patients with documented psychiatric comorbidities and 54 patients without psychiatric diagnoses (Figure 1).

Baseline demographic and clinical characteristics were generally comparable between groups (Table 1). The mean age was 56 years in the psychiatric group and 58 years in the non-psychiatric group. The proportion of female patients was slightly higher in the psychiatric group (61%) compared to the non-psychiatric group (55%). Mean pain duration was similar between groups (5.2 vs. 4.8 years).

**Table 1:** Baseline demographics and clinical characteristics. Continuous variables were analyzed using an independent-samples Student’s t-test. Categorical variables were analyzed using chi-square testing. NRS: Numeric Rating Scale

Characteristic	Psychiatric Group (n = 46)	Non-psychiatric Group (n = 54)	Test Statistic	df	p-value
Mean age (years)	56	58	t = 1.12	98	0.26
Female sex (%)	61	55	χ^2^ = 0.38	1	0.54
Baseline NRS pain score, mean	7.8	7.6	t = 0.74	98	0.46
Mean pain duration, years	5.2	4.8	t = 0.91	98	0.36

Baseline NRS pain scores were also comparable between groups (7.8 vs. 7.6). At three-month follow-up, patients without psychiatric comorbidities demonstrated significantly greater improvement, with lower mean NRS pain scores (4.2 vs. 5.7) and greater reduction in pain scores (mean reduction: 3.4 vs. 2.1, p = 0.01) (Table 2).

**Table 2:** Pain and functional outcomes at the three-month follow-up. Continuous variables were compared using an independent-samples Student’s t-test. Categorical variables were analyzed using chi-square testing. NRS: Numeric Rating Scale

Outcome	Psychiatric Group (n = 46)	Non-psychiatric Group (n = 54)	Test Statistic	df	p-value
Baseline NRS, mean ± SD	7.8 ± 1.2	7.6 ± 1.1	t = 0.74	98	0.46
Three-month NRS, mean ± SD	5.7 ± 1.4	4.2 ± 1.3	t = 5.31	98	<0.001
Mean NRS reduction	2.1 ± 1.1	3.4 ± 1.2	t = 2.61	98	0.01
Patients reporting functional improvement (%)	48	72	χ^2^ = 5.94	1	0.01

Functional improvement was significantly more common among patients without psychiatric comorbidities compared to those with psychiatric conditions (72% vs. 48%, χ^2^ = 5.94, p = 0.01). Similarly, opioid reduction was significantly more common in the non-psychiatric group (56% vs. 32%, χ^2^ = 5.76, p = 0.02) (Table 3).

**Table 3:** Functional and opioid-related outcomes at the three-month follow-up. Categorical variables were analyzed using the chi-square test.

Outcome	Psychiatric Group (n = 46)	Non-psychiatric Group (n = 54)	Test Statistic	df	p-value
Improved function (%)	48	72	χ^2^ = 5.94	1	0.01
No change in function (%)	35	20	χ^2^ = 2.87	1	0.09
Worsened function (%)	17	8	χ^2^ = 1.89	1	0.16
Reduced opioid use (%)	32	56	χ^2 ^= 5.76	1	0.02
No change in opioid use (%)	50	34	χ^2^ = 2.66	1	0.10
Increased opioid use (%)	18	10	χ^2^ = 1.41	1	0.23

## Discussion

The findings of this retrospective cohort study demonstrate that patients with baseline psychiatric comorbidities experienced significantly less clinical improvement following interventional pain procedures compared with patients without psychiatric diagnoses. Specifically, individuals with psychiatric conditions demonstrated smaller reductions in NRS pain scores, lower rates of functional improvement, and reduced likelihood of opioid reduction at three-month follow-up. These findings further support the growing evidence that psychological and psychiatric factors substantially influence chronic pain outcomes and procedural responsiveness.

The observed association between psychiatric comorbidities and poorer pain outcomes is biologically plausible and supported by prior literature. Depression, anxiety, and PTSD have been linked to alterations in central pain modulation pathways, increased neuroinflammatory activity, dysregulation of serotonergic and noradrenergic neurotransmission, and enhanced central sensitization [6-8]. These neurobiological changes may amplify nociceptive processing and reduce responsiveness to interventional therapies that primarily target peripheral pain generators. Functional neuroimaging studies additionally demonstrate overlapping neural circuitry between chronic pain and affective disorders, including involvement of the anterior cingulate cortex, insula, hippocampus, and prefrontal cortex [10].

Psychological factors may also influence procedural outcomes through behavioral and cognitive mechanisms. Patients with depression or anxiety frequently exhibit maladaptive coping strategies, pain catastrophizing, fear-avoidant behaviors, reduced self-efficacy, and impaired adherence to rehabilitation programs [19,21]. These factors may contribute to persistent disability despite technically successful interventional procedures. Furthermore, psychiatric comorbidities are associated with impaired sleep quality, decreased physical activity, and social isolation, all of which may worsen chronic pain severity and interfere with recovery [22].

Our findings are consistent with prior investigations demonstrating worse pain-related outcomes among patients with psychiatric disorders undergoing pain-related interventions. Previous studies have reported that depression and anxiety are associated with poorer outcomes after lumbar spine surgery, spinal cord stimulation, radiofrequency ablation, and opioid-based pain management strategies [14-18]. Similarly, psychosocial distress has been identified as an independent predictor of diminished functional recovery and lower patient satisfaction across multiple chronic pain treatment modalities [23].

**Figure 2:**
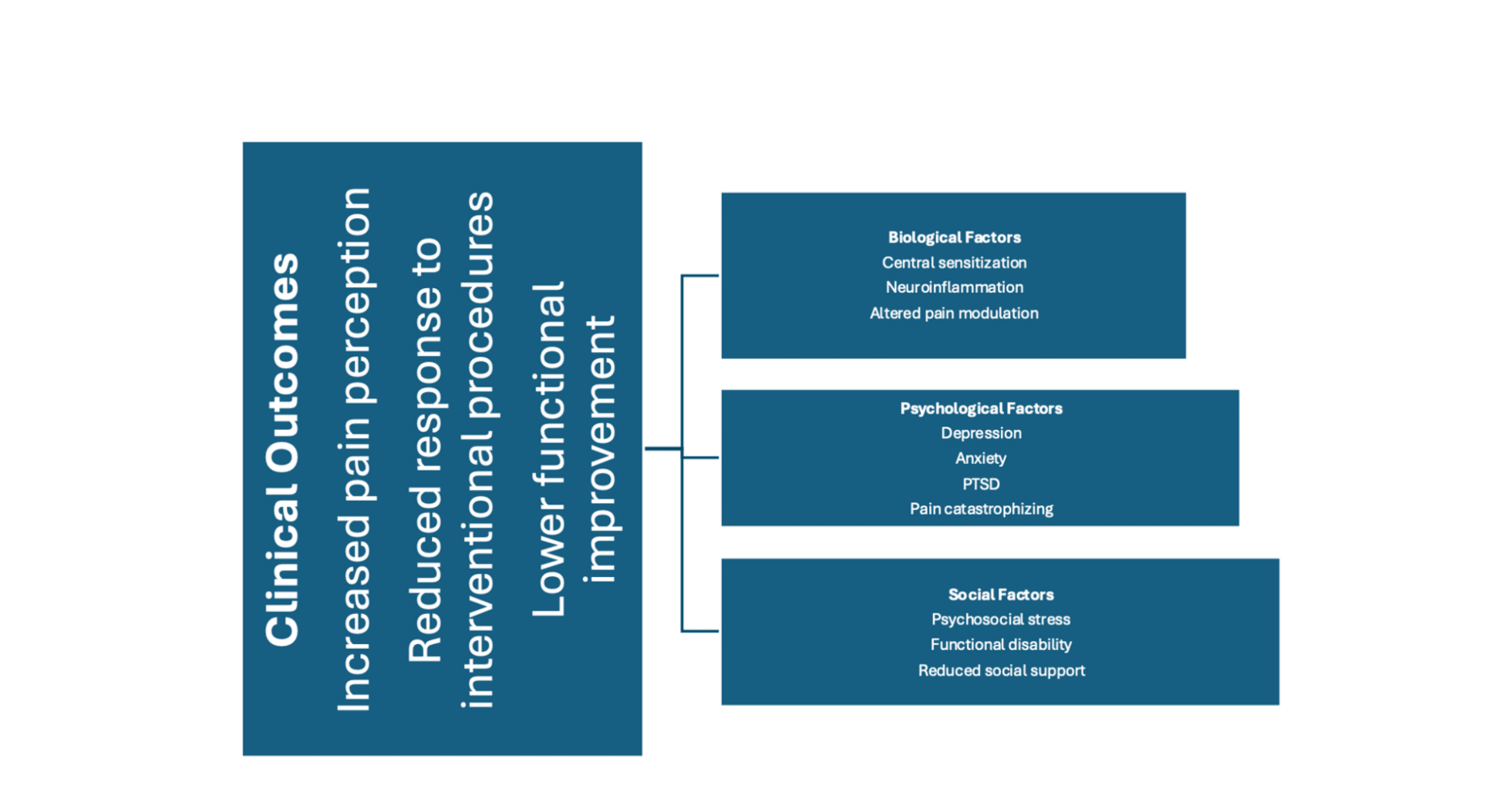
Biopsychosocial model of pain illustrating factors contributing to clinical outcomes. This schematic demonstrates the interaction between biological, psychological, and social factors in influencing pain perception and treatment response. Biological mechanisms such as central sensitization, neuroinflammation, and altered pain modulation, together with psychological factors including depression, anxiety, post-traumatic stress disorder (PTSD), and pain catastrophizing, as well as social determinants such as psychosocial stress, functional disability, and reduced social support, contribute to increased pain perception, reduced response to interventional procedures, and lower functional improvement. Image Credit: The image was created by the authors using Microsoft PowerPoint (Microsoft Corp., Redmond, WA, USA).

Importantly, the results of this study further reinforce the clinical relevance of the biopsychosocial model of pain. Contemporary pain medicine increasingly recognizes that successful treatment outcomes require more than procedural intervention alone. Comprehensive pain management strategies integrating psychological assessment, behavioral therapy, psychiatric optimization, physical rehabilitation, and patient education may improve overall treatment responsiveness and long-term outcomes [4,24]. Screening tools evaluating depression, anxiety, catastrophizing, and psychosocial stressors may help identify high-risk patients prior to interventional procedures and facilitate individualized multidisciplinary treatment planning. These findings further support the biopsychosocial model of pain, in which biological, psychological, and social factors interact to influence pain perception and treatment outcomes (Figure 2).

The present study additionally demonstrated lower rates of opioid reduction among patients with psychiatric comorbidities. This observation aligns with prior evidence showing increased opioid dependence, opioid misuse risk, and prolonged opioid utilization among patients with depression and anxiety disorders [25]. Persistent psychological distress may contribute to ongoing pain amplification and reduced procedural efficacy, thereby limiting opportunities for opioid tapering following intervention.

Several limitations should be considered when interpreting these findings. First, the retrospective design limits causal inference and introduces potential selection bias. Second, psychiatric diagnoses were identified through medical record documentation and may underestimate the true prevalence or severity of psychiatric illness. Third, variability in procedural type, pain etiology, and concurrent treatments may have influenced outcomes. Additionally, the study did not include standardized psychometric instruments such as the Patient Health Questionnaire-9 (PHQ-9), Generalized Anxiety Disorder-7 (GAD-7), or Pain Catastrophizing Scale assessments, which may provide more granular characterization of psychological burden. Finally, the relatively modest sample size and single-center design may limit generalizability.

Future prospective multicenter studies with standardized psychiatric assessments, longer follow-up periods, and multivariable analyses are needed to further clarify the relationship between psychiatric comorbidities and procedural outcomes in interventional pain medicine. Additional investigation into integrated behavioral and interventional treatment models may also help optimize outcomes in this complex patient population.

Several limitations should be considered when interpreting these findings. First, the retrospective design limits causal inference and introduces potential selection bias. Second, psychiatric diagnoses were identified through documented clinical records and may underestimate the true prevalence or severity of psychiatric illness. Additionally, the retrospective design did not allow a reliable determination of whether psychiatric illness preceded or followed chronic pain onset. Psychiatric diagnoses were identified through documented clinical records rather than standardized Diagnostic and Statistical Manual of Mental Disorders, Fifth Edition (DSM-5)-based assessments, and detailed information regarding psychiatric treatment modalities, medication use, psychotherapy participation, and treatment adherence was not consistently available for analysis.

## Conclusions

Baseline psychiatric comorbidities were associated with significantly reduced clinical improvement following interventional pain procedures, including smaller reductions in pain scores, lower rates of functional recovery, and decreased opioid reduction. These findings support the importance of the biopsychosocial model in chronic pain management and highlight the potential value of incorporating psychological assessment, psychiatric optimization, and multidisciplinary treatment strategies into interventional pain care. Future prospective studies are needed to further define the impact of psychiatric illness on procedural outcomes and identify targeted interventions that may improve treatment response in this population.
